# Dealing with the data deluge in high throughput screening

**DOI:** 10.1155/S1463924600000237

**Published:** 2000

**Authors:** Philip Skehan

**Affiliations:** Andes Pharmaceuticals Inc. 26520 Northeast 15th Street Redmond WA 98053 USA

## Abstract

Numerical taxonomy and pattern recognition analysis offer powerful tools that can greatly reduce the information burden of multiple-assay screening programs. These methods can be used to rationally design prescreens, identify assays that have similar chemical response patterns, select reporter assays for chemical response groups, evaluate drug selectivity, and predict a drug's likely mechanism of action. When combined with assays designed to identify lead compounds that have characteristics likely to cause failure at a later and more expensive stage of development, a simple three-stage primary discovery process consisting of a rational prescreen, reporters, and clinical failure assay can reduce the number of required culture wells by more than 20-fold and can eliminate all but 1–2 drugs per 1000 tested as leads for further evaluation and development.


Journal of Automated Methods & Management in Chemistry, Vol. 22, No. 5 (September–October 2000) pp. 145–148

### Dealing with the data deluge in high throughput screening

**Philip Skehan,**

*Andes Pharmaceuticals, Inc., 26520 Northeast 15th Street, Redmond, WA 98053, USA*

*Numerical taxonomy and pattern recognition analysis offer powerful tools that can greatly reduce the information burden of multiple-assay screening programs. These methods can be used to rationally design prescreens, identify assays that have similar chemical response patterns, select reporter assays for chemical response groups, evaluate drug selectivity, and predict a drug's likely mechanism of action. When combined with assays designed to identify lead compounds that have characteristics likely to cause failure at a later and more expensive stage of development, a simple three-stage primary discovery process consisting of a rational prescreen, reporters, and clinical failure assay can reduce the number of required culture wells by more than 20-fold and can eliminate all but 1–2 drugs per 1000 tested as leads for further evaluation and development.*

### Introduction

The extraordinary volume of data generated by high throughput screening has shifted the bottlenecks in drug discovery from compound acquisition and screening to the management and analysis of data. This presentation explores two questions. How can biological data be used to make the screening process smaller, simpler, faster, and cheaper? And how can biological data be used to better prioritize lead compounds for further development? Numerical taxonomy and pattern recognition offer powerful tools for addressing these questions, and can greatly reduce the information burden of multi-assay screening programs.

### Identifying chemical response groups

Hundreds of different drug discovery assays are now available. With cancer, there are more than 300 different human neoplastic diseases, each a potential screening target. Do we need to screen against all of them? Or, is it possible that some cancers may be similar enough to one another in their chemical response patterns that a single assay might serve as a reporter for an entire group of cell lines?

To explore this issue, we performed similarity analyses (Pearson's and Kendall's tau) and three types of cluster analysis (city block, Pearson's coefficient, and Kendall's tau taxonomic distance metrics using both average and median linkage methods for each) of the chemical response patterns for 72 tumour lines in tissue culture [1, 2]. Most of the lines were of human origin, but some animal lines were used as well. Nearly 400 bioactive compounds were screened against the cell lines in dose–response mode using a homogeneous propidium iodide assay that measures the fluorescence emission of dye molecules intercalated into double stranded regions of RNA and DNA [3]. The compounds encompassed a wide range of chemical structures and mechanisms of action.

As the cell lines employed had markedly different doubling times, conventional methods for quantifying drug effects, such as $I_{50}$ or $T/C$, could not be used because the biological significance of such indices is proportional to growth rate. Instead, we calculated a response function $R_F$ that allows efficacy and potency to be compared for cell lines with very different growth rates [4, 5]. Three measurements must be made to calculate $R_F$: a time zero sample $Z$ at the start of an assay, and end of assay control $C$ and test $T$ samples. If growth of the test cultures is non-negative ($T \geqslant Z$), $R_F$ is calculated as $100[(T-Z)/(C-Z)]$. If $T < Z$, then $R_F$ is calculated as $100[(T-Z)/Z]$. Control cultures have an $R_F$ value of $+100$, total net growth inhibition has an $R_F$ value of 0, and an $R_F$ value of $-100$ reflects total culture extinction. $R_F$ values greater than 100 signify a net growth stimulation; an $R_F$ value between 0 and 100 represents net growth inhibition, and a value between 0 and $-100$ indicates net cell killing.

For each compound, the test concentration was identified that produced maximum differential activity as indexed by the mean absolute deviation of efficacy values for all cell lines. At this concentration, a selectivity coefficient $S_E = \langle R_F \rangle - R_F$ was calculated for each cell line by subtracting the efficacy value of a cell line $R_F$ from the median efficacy value $\langle R_F \rangle$ of all cell lines.

Efficacy rather than potency was used for three reasons. First, potency is an interpolation while efficacy is a measured value. Second, potency requires an arbitrary activity criterion, such as total growth inhibition ($R_F = 0$), which is often not achieved with a fixed assay protocol; when the activity criterion is not achieved, potency values must be either deleted from the database or assigned an artificial value such as the highest or lowest concentration tested. Assigned values can be in error by orders of magnitude. Third, it is not uncommon for the dose–response curve of one assay to just barely achieve the activity criterion and the curve for another assay to just barely fail to do so. Even though the two curves are nearly superimposable, very large apparent differences in potency can arise that are artefactual.

Ten different chemical response groups were identified, each with its own unique chemical response pattern. The number of cell lines in each group ranged from two to 16 with a median of 5.5. There were four cell lines that were related to one or another of the ten groups, but not strongly enough to meet the inclusion criterion, and there were seven cell lines whose chemical response patterns were unrelated to any of the ten response groups. The results of the cluster and similarity analyses were essentially identical, suggesting that the taxonomic conclusions reached are robust.

Of the ten chemical response groups, six were considered to be clinically relevant, while four were not. The six clinically relevant groups varied considerably in their sensitivity to the chemical screening library. Maximum group median selectivity coefficients ranged from a low of 62 to a high of 150. Similarly, the most resistant group was selectively sensitive to just 79 compounds, while the most sensitive group selectively responded to 271.

### Reporter assays

The question now arose: is it necessary to screen against all of the cell lines within a response group, or might there exist a single cell line that can serve as a reporter for the entire group? For each chemical response group, compounds that were selectively active against the group were rank ordered by median group selectivity coefficient $S_E$ from the highest selectivity to zero selectivity. The cell line was then identified that recognized the greatest number of compounds active at various selectivity levels ranging from 0 to over 100. $S_E$ values from 0 to 40 represent weak selectivity; from 40 to 80 represent moderate selectivity; and greater than 80 represent strong selectivity.

Four of the reporter lines had an accuracy of better than 90% at $S_E$ values of 20 or greater, and five exceeded 90% at an $S_E$ value of 40 or greater (figure 1). The worst behaving reporter line displayed an accuracy of more than 80% at $S_E$ values of 40 or greater. These findings indicate that single reporter assays can reflect the behavior of an entire chemical response group with reasonable accuracy, and the use of reporter assays can greatly reduce the screening burden of multi-assay screening programs. The six reporter lines comprise the basis for a selective toxicity screen, which operates in dose–reponse mode.

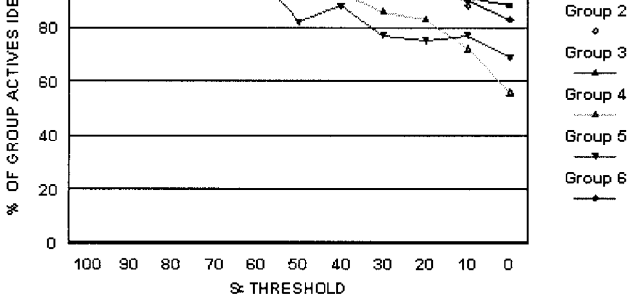

*Figure 1. Accuracy of reporter lines.*

### Rationally designed prescreen

The identification of reporter lines allowed us to reduce our number of cancer assays by more than tenfold. However, about 85% of the compounds tested were inactive, with the result that a further reduction in the screening burden could be achieved if a prescreen with fewer than six cell lines could be constructed.

Prescreens can be rationally designed by statistically determining the minimum number of assays required to identify at a specified level of accuracy those compounds active against one or more of a larger group of screens. Compounds were tested at a single high dose, and 50% net growth inhibition was used as an activity criterion for the prescreen analysis. The entire panel of cancer cell lines was examined to determine the single cell line that correctly identified the greatest number of compounds that were active against five or more cell lines (noise level of the system). That cell line was placed in the prescreen. The remaining cell lines were then examined to find the next cell line that correctly identified the greatest number of active compounds not already identified by the first cell line. That cell line was also placed in the prescreen. This process was repeated iteratively until a prescreen was developed that could predict activity within the entire panel of lines with an accuracy of greater than 95%.

Surprisingly, this criterion was achieved with just two cell lines. The two-cell line prescreen identified the activity of more than 800 compounds with an accuracy of 95.5%. There were 0.7% false positive and 3.8% false negative identifications.

### Clinical failure assays

A common failing of many discovery screens is their inability to identify lead compounds that have characteristics likely to cause failure at a later and more expensive stage of development. Simple *in vitro* assays predictive of likely clinical failure can often be developed and included as part of the primary drug discovery process. Such clinical failure assays can quickly eliminate all but a few competing leads from further development. With cell-based screening, a clinical failure assay combined with a rational prescreen and response group reporters can eliminate all but 1–2 drugs per 1000 tested as leads for further evaluation and development.

One of the major reasons for the clinical failure of anticancer drugs is the survival of residual tumour burden. Most traditional anticancer drugs act more as growth inhibitors than as target-eradicating cytotoxins. They may well kill a portion of a tumour cell population, but leave surviving cells that can grow back to life threatening proportions.

To identify drugs likely to permit the survival of residual tumour burden, we developed a long term recovery (LTR) assay that was incorporated as the third leg of our primary discovery process [3]. In the LTR assay, cultures are incubated with test compounds for 48 hours in T25 flasks at half-, just-, and supra-maximal concentrations. The drugs are then removed and the cultures washed and fed with a fresh drug-free non-bicarbonated growth medium that uses beta-glycerophosphate as a buffer. This medium, e.g. Gibco's Nonbicarbonated Growth Medium, is pH stable under atmospheric conditions, does not require a $CO_2$-enriched environment, and contains phenol red as a colorimetric visual pH indicator [6]. The flasks are capped tightly, and placed in a 37 °C incubator, where they are incubated for 60 days or until cellular regrowth is obvious.

There are two end points in the LTR assay: metabolic and proliferative. Metabolic recovery is monitored by visual inspection three times a week. Where metabolically surviving cells remain, their secretion of organic acids gradually changes the colour of the growth medium's pH indicator dye from red to reddish-orange, to orange and finally to yellow. When a colour change has become obvious, cultures are inspected microscopically to confirm that the metabolism is the result of cellular regrowth and not microbial contamination. The extent of the pH change can be quantitated by measuring the phenol red optical density at 560 nm. Proliferative recovery is quantitated by sulphorhodamine B protein optical density or the propidium iodide fluorescence from double stranded RNA and DNA dye intercalation [3]. Control flasks are collected at the time of drug addition and at the end of the 48 hour incubation period. Test samples are collected at these same times and after recovery has occurred. $R_F$ values are calculated as described above.

In the LTR assay, 85% of high priority lead compounds emerging from the selective toxicity screen failed to completely erradicate tumour cells in culture, allowing the cells subsequent regrowth. With most of the drugs that failed the LTR assay, regrowth was obvious within 3–5 days and sometimes within 1–2 days.

### Three-stage primary screening process

The primary screening process that Andes has adopted consists of three stages: (1) an initial prescreen of two cell lines with single high-dose testing; (2) a selective toxicity screening panel in which test samples are screened in dose–response mode against six reporter lines; and (3) a long term recovery assay. For every 1000 compounds that enter the screening process, about 150 are active in the prescreen, 11 exhibit moderate or stong selectivity in the selective toxicity screens, and 1.7 show no regrowth in the LTR assay. The overall percentage of compounds that progress to further development is about 0.17% (figure 2).

### Multi-assay fingerprints

Multi-assay screening data can be visually represented as bar graph fingerprints projecting to either the right or left of a central reference value depending on whether an assay is more or less sensitive to a test compound than is the reference value. With mean graphs [7], log potencies are first determined for each assay, and the mean log potency identified. The mean log potency is then sub-

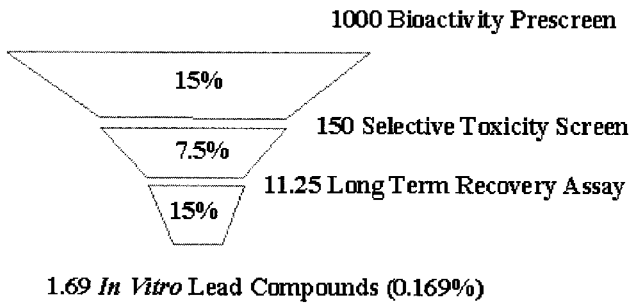

*Figure 2. The Andes cancer screening funnel.*

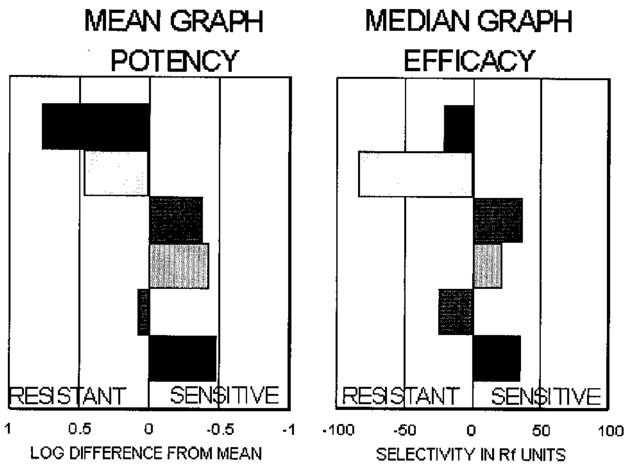

*Figure 3. Potency and efficacy fingerprints.*

tracted from each individual value to produce an index of differential sensitivity to the test compound. Negative values of this difference indicate that an assay is more sensitive than the mean, while positive values reflect resistance.

Similarly, an efficacy fingerprint can be constructed by first finding the median efficacy for a set of $R_F$ values, then subtracting individual $R_F$ values from this median. Again, individual differences from the median are plotted as bars projecting to the right for assays that are more sensitive and to the left for assays that are more resistant than the median.

Multi-assay fingerprints, first used widely by the National Cancer Institute, have proven valuable in a number of ways. They provide a simple method for presenting complicated data in a manner that is easily understood and which visually highlights patterns of differential sensitivity (figure 3).

### Comparing fingerprints

Fingerprints can be compared both visually and numerically. The mean absolute difference (MAD) of two fingerprints provides a useful quantitative index of their similarity or difference. Two compounds are compared by summing the absolute difference of either their $R_F$ or log potency values for each assay, then dividing this sum by the number of assays. The more similar two compounds are, the smaller their MAD will be.

When the fingerprint of a test compound is compared with the fingerprints of a large number of previously tested drugs, a great deal of useful information can be obtained. The best database matches often suggest the likely mechanism of action of a new test compound. Similarly, structural analogues are often very close matches as well, and can suggest likely chemical structures of active components in crude natural product extracts. Even when close matches are not analogues, they sometimes possess similar molecular surface properties or three-dimensional conformations. The insight that these similarities provides can be valuable in rational drug design. Finally, fingerprint comparisons are extremely useful in studies of structure-activity relationships.

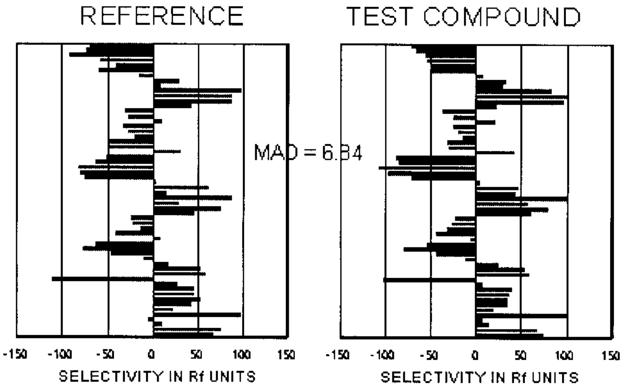

*Figure 4. Comparing fingerprints.*

### Association coefficients

Drug response patterns can also be qualitatively compared using association coefficients such as the simple matching coefficient $S_M$ and Jacard's similarity coefficient $S_J$. These association coefficients compare two drugs by determining on an assay by assay basis whether the drugs are matched ($a = 11$ or $d = 00$) or mismatched ($b = 01$ or $c = 10$) in their effects, where 1 represents activity and 0 inactivity.

$S_M$ is well suited to the situation in which active and inactive outcomes are roughly equal in frequency. It is the ratio of matches to matches plus mismatches: $S_M = (a + d)/(a + b + c + d)$. $S_J$ ignores the doubly inactive matches (00), and is well suited to the situation in which active compounds are infrequent by comparison with inactive: $S_J = a/(a + b + c)$. A variety of other association coefficients are in common use as well [1, 2]. Association coefficients can be used for the same purposes as fingerprint comparisons, or can be used in conjunction with fingerprints. For the two compounds shown in figure 4, $S_J$ was 0.903 for selectivity values ($S_E$) greater than 0, and $S_J$ 0.714 for selectivity values greater than 50.

Association coefficients have a particularly useful property: they can be used to compare multistate characteristics defined by logic operators such as AND, OR, and NOT. Thus an element for comparison could be: active in assays 1–10 NOT active in assays 11–15 AND water soluble at 1 mM AND a hydrophobic interior AND(sensitive to antimicrotubulars OR antifols). Such complex activity criteria permit very sophisticated comparisons to be performed.

### References

1. Pielou, E. C., 1984, *The Interpretation of Ecological Data.* (Chichester, UK: John Wiley & Sons).
2. Sneath, P. H. A. and Sokal, R., 1973, *Numerical Taxonomy*, (New York: W. H. Freeman & Co).
3. Skehan, P., 1995, Assays of cell growth and cytotoxicity. In G. P. Studzinski (ed.), *Cell Growth and Apoptosis*, (Oxford: IRL Press at Oxford University Press), pp. 169–191.
4. Skehan P, Thomas, J. and Friedman, S. J., 1986, Postconfluency MDCK monolayers as in vitro models of solid tumour chemosensitivity, *Cell Biology and Toxicology*, **2**, 357–368.
5. Monks, A., Scudiero, D., Skehan, P., Shoemaker, R., Paull, K., Vistica, D., Hose, C., Langley, J., Cronice, P., Vaigro-Wolff, A., Gray-Goodrich, M., Campbell, H., Mayo, J. and Boyd, M. R., 1991, Feasibility of a high flux anticancer drug screen utilizing disease-oriented panels of human tumour cell lines in culture. *Journal of the National Cancer Institute*, **83**, 757–766.
6. Vistica D., Scudiero, D., Skehan, P., Monks, A. and Boyd. M. R., 1990. New carbon dioxide-independent basal growth medium for cultures of diverse tumour and nontumour cells of human and nonhuman origin. *Journal of the National Cancer Institute*, **82**, 1055–1061.
7. Paull, K. D., Shoemaker, R. H., Hodes, L. *et al.* 1989. Display and analysis of patterns of differential activity of drugs against human tumour cell lines. *Journal of the National Cancer Institute*, **81**, 1088–1092.

